# Research progress on long non-coding RNAs for spinal cord injury

**DOI:** 10.1186/s13018-023-03989-x

**Published:** 2023-07-22

**Authors:** Musen Zhong, Guangya Fan, Zhongcheng An, Chen Chen, Liqiang Dong

**Affiliations:** 1 The Second School of Clinical Medicine, Zhejiang Chinese Medical University, Hangzhou, China; 2 Orthopedic Traumatology II, The Sceond Affiliated Hospital of Zhejiang Chinese Medical University, Hangzhou, China

**Keywords:** Long non-coding RNA, Spinal cord injury, Mechanism of action, Signaling pathway

## Abstract

Spinal cord injury is a complex central nervous system disease with an unsatisfactory prognosis, often accompanied by multiple pathological processes. However, the underlying mechanisms of action of this disease are unclear, and there are no suitable targeted therapeutic options. Long non-coding RNA mediates a variety of neurological diseases and regulates various biological processes, including apoptosis and autophagy, inflammatory response, microenvironment, and oxidative stress. It is known that long non-coding RNAs have significant differences in gene expression in spinal cord injury. To further understand the mechanism of long non-coding RNA action in spinal cord injury and develop preventive and therapeutic strategies regarding spinal cord injury, this review outlines the current status of research between long non-coding RNAs and spinal cord injury and potential long non-coding RNAs targeting spinal cord injury.

## Introduction

Spinal cord injury (SCI) is a disabling, prolonged, and costly central nervous system disease [1, 2]. According to relevant literature statistics [3]: Nationwide, the number of SCI patients was about 0.9 per 1 million in 2019. It is prevalent in elderly males, mainly caused by falls and traffic accidents. As the population gets older and older, the incidence of SCI rises year by year, leading to an increased economic burden, and reduced quality of life is gradually being taken into account. External forces cause primary spinal cord injury. In contrast, secondary spinal cord injury is caused by permanent damage to normal neuronal cells due to ischemia and hypoxia, electrolyte disturbance, edema, accumulation of excitotoxic neurotransmitters, inflammatory response, and oxidative stress [4]. Although many experimental studies have been conducted to treat SCI, the therapeutic effect of interventions implemented for this purpose is limited, so how to effectively treat SCI is still a severe test.

Non-coding RNA (NCRNA) is RNA that does not encode into proteins. It mainly includes transfer RNA (tRNA), ribosomal RNA (rRNA), and small nuclear RNA (snRNA), as well as regulatory ncRNAs, including PIWI-interacting RNA (piRNA), long non-coding RNA (lncRNA) and microRNAs (miRNAs), and other unknown types [5]. It has been proposed that some miRNAs regulate the tendon healing process by participating in biological processes such as oxidative stress, tendon collagen formation, and cellular senescence in damaged tendons [6]. Some miRNAs can intervene in osteoarthritis development by regulating the level of inflammatory factors in osteoarthritis, and it is conjectured that the severity of osteoarthritis can be determined by the expression level of related miRNAs [7]. Some miRNAs may also regulate disease trends by participating in several pathological processes such as apoptosis, oxidative stress, and inflammatory responses in spinal cord ischemia–reperfusion injury [8]. However, lncRNAs and miRNAs are not independent, and their relationship is inextricably linked. It has been found that lncRNAs can compete with miRNAs to regulate the expression of downstream genes, forming an endogenous RNA (ceRNA) regulatory network involved in various pathological processes [9].

In recent years, some related studies have found that lncRNA is involved in developing many disease processes. lncRNA is differentially expressed genetically in Parkinson’s, cancer, ischemic stroke, and other diseases. For example, differential expression of lncRNA and mRNA expression profiles in Parkinson’s revealed that the expression level of lnc-MKRN2-42:1 was positively correlated with the severity of dyskinesia and dysarthria [10], lncRNA can promote tumor cell proliferation by regulating the expression of oncogenes, binding to miRNAs, and influencing the regulation of miRNAs [11]. The lnc-D63785 knockdown can induce miR-422a accumulation in ischemic stroke and aggravate neuronal apoptosis [12]. Alterations in gene expression play an essential role in the pathogenesis of secondary spinal cord injury. However, the mechanism of the action of lncRNAs regulating SCI-related gene expression is unclear.

LncRNA is characterized as having a length of more than 200 nucleotides. RNA polymerase II transcribes the majority of lncRNAs, and certain lncRNAs can be stabilized by polyadenylation at the 3' end and encapsidation at the 5′ end [13]. LncRNAs regulate biological processes through different mechanisms of action [14]. Wang, K. C et al. [15] divide the interaction between lncRNA and DNA, RNA, and proteins into four types: 1. Signal lncRNAs: lncRNAs act as signaling molecules, affecting the response of molecules such as signaling pathways and regulating downstream gene transcription; 2. Inducible lncRNAs: Inducible lncRNAs control transcription by interacting with transcription factors. lncRNAs interact with transcription factors and chromatin-binding proteins to prevent them from binding to target genes, an ability also known as a molecular sponge; 3. Guide lncRNAs: guide-related protein complexes to bind to specific target genes; and 4. Scaffold lncRNAs: lncRNAs serve as the central platform for assembling various protein complexes and regulating intermolecular interactions.

It has recently been shown that SCI induces changes in lncRNA gene expression and that lncRNA plays a critical role in SCI [16, 17]. Therefore, an in-depth understanding of the role of lncRNAs in SCI can help to develop a treatment plan for SCI. This review outlines our present understanding of the regulatory mechanisms of SCI lncRNA treatment.

## Pathogenesis of spinal cord injury

**Fig. 1:**
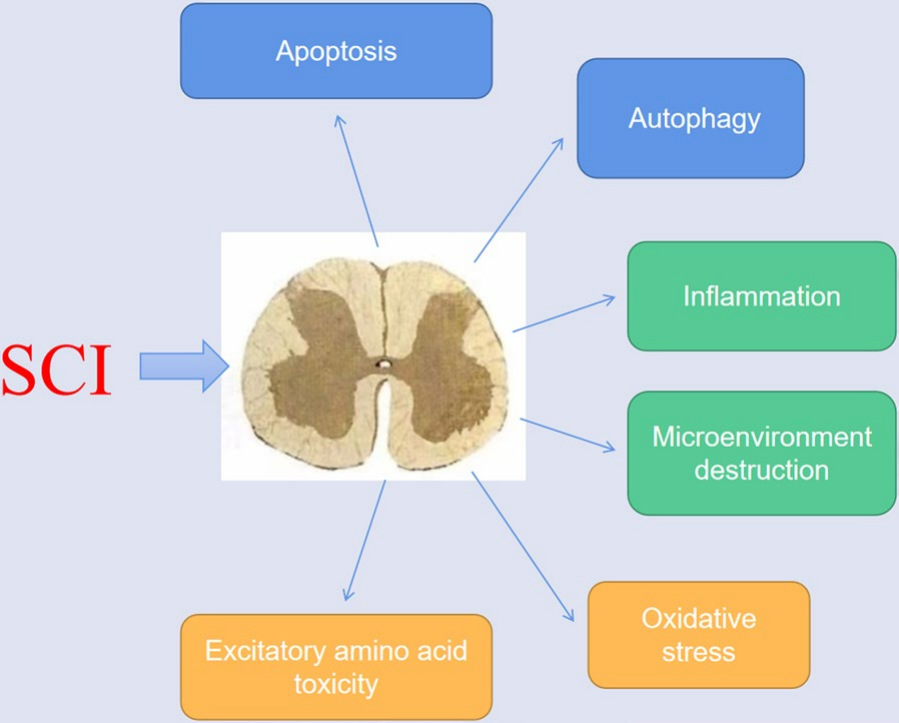
Pathological mechanisms after spinal cord injury

SCI is mainly divided into primary and secondary injuries caused by various factors. Among them, apoptosis and autophagy, inflammation, microenvironment, oxidative stress, excitatory amino acid toxicity, and other pathological processes play an essential role in SCI (Fig. 1).

### Apoptosis and autophagy

Apoptosis is a form of programmed cell death that maintains the stability of the internal environment in the body by removing non-functional, abnormal, and harmful cells. Neurons and oligodendrocytes in SCI undergo widespread apoptosis, leading to neurological dysfunction, and it can exacerbate SCI [18]. Cysteinyl aspartate-specific proteinase (Caspase), Bax, and Bcl-2 genes are intimately involved in the biological mechanisms associated with apoptosis [19]. Autophagy is an important defense and protection mechanism in the organism. Autophagy maintains metabolic homeostasis in the body mainly by degrading and removing damaged and denatured proteins and lost organelles. There are three primary forms of autophagy: macroautophagy, microautophagy, and molecular chaperone-mediated autophagy, which ultimately transports substances to lysosomes for degradation and recycling. Autophagy is controlled by a number of mechanisms, including the serine/threonine protein kinase ULK1 complex, the Beclin1 complex, positive AMPK regulation, and negative mTOR target regulation [20]. Some related studies have reported [21] that autophagy promotes neurological recovery, reduces neuronal cell apoptosis, and suppresses inflammation. On the other hand, excessive autophagy can lead to neuronal cell death. Therefore, proper activation of autophagy can help improve SCI.

### Inflammation

After spinal cord injury, the integrity of the blood–spinal cord barrier (BSCB) is disrupted, leading to the infiltration of peripheral immune cells into the site of injury, producing many pro-inflammatory cytokines involved in the inflammatory response, and affecting the prognosis of SCI. Among these immune cells, neutrophils are the first to infiltrate the damaged part of the spinal cord and reach their peak within 24 h. It can have a bactericidal effect through phagocytosis and clearance and an indirect tissue repair effect. However, it has also been shown that as neutrophils accumulate, they gradually produce products such as proteases, oxidases, and tissue degenerating enzymes that reverse the neurotoxicity of the stimulated neurons [22]. After this, microglia gradually enter the damaged area, and activated microglia can regulate NF-κB and IL-6 expression levels by activating the PI3K/Akt/mTOR signaling pathway and also upregulate TNF-α and IL-1 expression levels by activating STAT3. Large amounts of cytokines that inhibit the repair of nerves and synapses are released through multiple signaling pathways, thereby exacerbating the inflammatory response [23, 24]. It has been suggested that microglia in the injured area also have the role of scavenging neurotoxic substances and secreting nutritive substances to create good growth conditions for nerves and synapses [25]. In SCI, astrocytes, like microglia, are also a double-edged sword. On the one hand, activated astrocytes produce glial scarring to form a protective barrier, protecting the damaged area and inhibiting inflammatory cell activation and infiltration. On the other hand, astrocytes inhibit the self-repairing action of neuronal axons [26]. Astrocytes and microglia produce more pro-inflammatory factors after inflammatory stimulation exacerbating the inflammatory response process and leading to neuronal apoptosis [27, 28]. Lymphocytes also play an essential role in the inflammatory response, and lymphocytes can be divided into B lymphocytes and T lymphocytes. In SCI, due to changes in vascular permeability and the action of chemokines, lymphocytes are released from blood vessels and enter the area of injury to release large amounts of inflammatory factors (such as IFN-γ, TNF-α, and IL-2), which exacerbate the inflammatory cascade response and cause damage to the spinal cord microenvironment.

### Microenvironment destruction

Pathological processes such as intracellular calcium overload, lipid peroxidation, inflammatory response, and neuronal apoptosis occur after SCI, destabilizing the spinal cord microenvironment and exacerbating secondary spinal cord injury. Some related researchers [29] divided the microenvironmental changes into three levels: tissue, cellular, and molecular, according to the research perspective. Tissue level: The disruption of local capillaries and blood–spinal cord barrier (BSCB) caused by SCI promotes the increase in cytokines and chemokines, causes edema and ischemia in neural tissues, and forms glial scars that prevent axonal regeneration and growth. Cellular level: SCI promotes the differentiation of astrocytes, the loss of oligodendrocytes, and the transformation of microglia and macrophages into the M1 phenotype, which is detrimental to the recovery of SCI. Molecular level: Neurotrophic factors include brain-derived neurotrophic factor (BDNF), nerve growth factor (NGF), and neurotrophic factor (NT), and their precursors are disproportionately balanced; changes in cytokines and chemokines such as interleukin-1 (IL-1), IL-6, tumor necrosis factor-α (TNF-α), granulocyte–macrophage colony-stimulating factor (GM-CSF), and leukocyte inhibitory factor (LIF) and other cytokines and chemokines CXCL12, chemokine receptor CXCR4, and other chemokines are involved in the dynamic changes of the microenvironment after SCI; imbalance of sodium, potassium, and calcium ions can also lead to tissue cell edema, apoptosis, and axonal demyelination, which affect the recovery of spinal cord function.

### Oxidative stress

The imbalance between reactive oxygen species (ROS) and antioxidant levels after SCI leads to oxidative stress (OS), which includes hydrogen peroxide (H_2_O_2_), superoxide anion (O_2_^−^), and highly reactive hydroxyl radicals (HO^·^) that can damage neuronal cells and cause secondary spinal cord injury [30]. ROS can induce lipid peroxidation and attack and degrade polyunsaturated lipids in biofilms, leading to cell death, which disrupts calcium homeostasis, increased excitotoxicity of amino acids, and other processes that aggravate secondary spinal cord injury [31]. The degree of oxidative stress after SCI can be assessed by measuring the concentration of biomarkers such as glutathione (GSH), copper, zinc, superoxide dismutase (SOD), malondialdehyde (MDA), and acrolein [32].

### Excitatory amino acid toxicity

Glutamic acid (Glu) is a neurotransmitter of the central nervous system, and ischemia after SCI can induce an increase in extracellular Glu concentration. Appropriate Glu concentration maintains regular physiological activity. Excessive concentrations of Glu lead to increased permeability of sodium, potassium, and calcium ions in cell membranes, causing reactions such as increased Ca^2+^ and NO concentrations and activation of free radicals, resulting in toxic effects on the central nervous system [33, 34].

## Regulatory role of lncRNA in spinal cord injury

A large number of experimental studies have shown that there are significant differences in gene expression of lncRNAs in spinal cord injury. However, the mechanism of lncRNA action in spinal cord injury is complex and unclear. By extracting serum from SCI patients for in vitro experiments, some researchers found that lncRNA Mirt2 mediated the inflammatory response after SCI by inhibiting NF-κB and p38MAPK signaling pathways, suppressing apoptosis, and reducing the release of pro-inflammatory cytokines (e.g., TNF-α and IL-6) through sponge miR-429 [8]. In addition, other lncRNAs mitigate spinal cord injury in SCI by regulating apoptosis and autophagy, anti-inflammatory, improving microenvironment, and antioxidant pathways (Table 1) [35–37].

**Table 1:** List of studied lncRNA and their effect on SCI

lncRNA	Function	References
Mirt2	Suppressing apoptosis and reducing the release of pro-inflammatory cytokines	[34, 51]
LEF1-AS1	Accelerates apoptosis	[37]
XIST	Inhibiting microglia apoptosis and inflammatory response	[38]
MIAT	Reduces neuronal apoptosis	[39]
CasC7	Inhibit apoptosis	[40]
CCAT1	Alleviates apoptosis and inflammation	[41]
BDNF-AS	Exacerbating apoptosis and inflammatory responses	[43]
NEAT1	Alleviate inflammation	[44, 45]
Ftx	Inhibits the inflammatory response	[46]
Gm37494	Inhibits the inflammatory response	[47]
TUSC7	Inhibits microglial activation and inflammatory response	[48]
GBP9	Inhibits the inflammatory response	[49]
MALAT1	Exacerbating the inflammatory response	[50]
MEG3	Exacerbating the inflammatory response	[50]
ZNF667-AS	Inhibit the inflammatory response	[52]
DLEU1	Exacerbating the inflammatory response	[53]
SNHG4	Suppressed neuroinflammation	[54]
H19	Inhibits axonal regeneration	[55]
TUG1	Exacerbating the inflammatory response	[56]
Map2k4	Promote neuronal proliferation and inhibit apoptotic cell death	[57]
OL1	Promotes myelin formation	[58]
CASC9	Reduce SCI oxidative stress and inflammatory response	[59]
SOX2OT	Exacerbates oxidative stress, inflammatory response, and apoptosis	[60]
TCTN2	Alleviating oxidative stress, inflammatory response, and apoptosis	[61]
HOTAIR	Exacerbates oxidative stress, inflammatory damage, and neuronal apoptosis	[62]

### Regulation of apoptosis

SCI is often accompanied by apoptosis of neuronal cells. Many studies have found that many LNCRNAs are involved in the regulation of apoptosis in SCI such as lncRNA LEF1-AS1, lncRNA XIST, lncRNA MIAT, lncRNA CasC7, lncRNA CCAT1, and lncRNA BDNF-AS. lncRNA lymphatic enhancer factor 1 antisense RNA 1 (LEF1-AS1), a ceRNA of miR-222-5p, increases receptor activity-modifying protein 3 (RAMP3) by inhibiting the miR-222-5p expression and accelerates apoptosis [38]. LncRNA XIST sponges miR-27a and inhibits miR-27a gene expression. Knockdown of lncRNA XIST upregulates miR-27a to inhibit Smad ubiquitination regulatory factor 1 (Smurf1) expression, thereby inhibiting microglia apoptosis and inflammatory response [39]. LncRNA MIAT overexpression increases RBFOX2 protein levels by binding to RBFOX2 protein, promotes the production of anti-apoptotic factor MCL-1L, and reduces neuronal apoptosis [40]. Hydrogen sulfide is recognized as an essential neuromodulator in the central nervous system. Hydrogen sulfide inhibits miR-30c expression by upregulating lncRNA CasC7 levels to inhibit apoptosis [41]. The nuclear factor of activated T cells 5 (NFAT5) is a direct target negatively regulated by miR-218 to alleviate apoptosis and inflammatory injury. LncRNA CCAT1 was shown to be a ceRNA of miR-218, lncRNA CCAT1 overexpression alleviates apoptosis and inflammation through miR-218/NFAT5 signaling pathway, downregulation of Bax/Bcl-2 ratio and Caspase-3, as well as pro-inflammatory factors such as TNF-α, IL-1β, and IL-6 [42]. It has been shown [43] that miR-9-5p regulates the expression of monocyte chemotactic protein-inducible protein-1 (MCPIP1) expression and modulates macrophage activation and inflammatory responses, thereby regulating neuronal apoptosis. lncRNA BDNF-AS can target and inhibit the expression of miR-9-5p. Some researchers have used lithium to downregulate BDNF-AS, which counteracts the inhibition of miR-9-5p by lncRNA BDNF-AS inhibition, reducing SCI-induced apoptosis and inflammation [44].

### Regulation of neuroinflammation

The inflammatory response plays a crucial role in SCI. Some LncRNAs, such as ncRNA NEAT1, lncRNA Ftx, and lncGm37494, regulate inflammatory responses in SCI. LncRNA NEAT1 is the molecular sponge of miR-211-5p and BCL2L11, and the knockdown of lncRNA NEAT1 can reduce the production of inflammatory cytokines IL-6, IL-1β, and TNF-α through miR-211-5p/MAPK1 and miR-29a/BCL2L11 pathways and alleviate SCI inflammation [45, 46]. LncRNA Ftx competes for miR-382-5p and inhibits its gene expression to increase neuromodulin-1 (NRG1) expression and reduce the expression of inflammatory factors such as iNOS, IL-6, TNF-α, and IL-1β [47]. LncRNA Gm37494 converts microglia polarization from M1 to M2 by inhibiting miR-130b-3p and promoting PPARγ expression [48]. In addition, lncRNA TUSC7 inhibited microglial activation and expression of associated inflammatory factors by upregulating PPARγ by inhibiting miR-449a [49]. lncRNA GBP9 competes for binding miR-34a to counteract miR-34a inhibition of SOCS3, thereby regulating STAT1/STAT6 signaling and promoting macrophage polarization to M2 type [50]. Knockdown of lncRNA MALAT1 upregulated miR-199b to activate the IkappaB kinase-β (IKKβ)/nuclear factor κB (NF-κB) signaling pathway to alleviate SCI by inhibiting microglia inflammatory responses. LncRNA MEG3 is a ceRNA for miR-130a-5p. lncRNA MEG3 upregulates miR-130a-5p to increase CXCL12/CXCR4 expression and activates the TLR4/NF-κB signaling pathway, exacerbating the inflammatory response [51]. LncRNA Mirt2 protects PC12 cells from LPS-induced inflammatory damage by downregulating miR-429, thereby blocking NF-κB and p38MAPK signaling pathways [52]. The JAK-STAT signaling pathway is closely related to spinal cord neuronal death and ischemia. A related study reported that lncRNA ZNF667-AS could inhibit the inflammatory response and promote SCI recovery by inhibiting the JAK-STAT signaling pathway [53]. Serine/arginine protein kinase 1 (SRPK1), a downstream target of miR-133a-3p, is a multifunctional protein closely related to the inflammatory response. lncRNA DLEU1 competes to bind miR-133a-3p, counteracting the inhibitory effect of miR-133a-3p on SRPK1, upregulating IL-6, IL-1β, TNF-α, and other pro-inflammatory factor levels, and exacerbating the inflammatory response [54]. LncRNA SNHG4 suppressed neuroinflammation to relieve neuropathic pain by upregulating IL-6, IL-12, and TNF-α gene expression and downregulating IL-10 expression through the sponge miR-423-5p [55].

### Regulation of microenvironment

SCI produces a series of pathological responses that disrupt the microenvironment and affect the recovery of neuronal function. It has been found that lncRNA H19, lncRNA TUG1, lncRNA-Map2k4, and lncRNA OL1 can effectively improve SCI microenvironment damage. Knockdown of lncRNA H19 increased the expression of IGF1R and pS6, a marker of IGF1R-mediated activation of the mTOR pathway, promoting axonal regeneration and functional recovery in the spinal cord [56]. MiR-29b-1-5p restores lower limb motor function and alleviates BSCB injury after SCI by binding to MTDH while blocking the downstream NF-κB/IL-1β pro-inflammatory signaling pathway. lncRNA TUG1 is a ceRNA of MiR-29b-1-5p, and knocking down lncRNA TUG1 effectively improves the SCI microenvironment [57]. LncRNA plays a key role in central nervous system development and neurogenesis. FGF1, a downstream target gene of miRNA-199a, is distributed in the CNS and has a trophic support role for neurons. lncRNA-Map2k4 upregulates FGF1 expression by repressing miRNA-199a to promote neuronal proliferation and inhibit apoptotic cell death [58]. Oligodendrocytes are intimately involved in myelin formation in the CNS, and it has been found that lncRNA OL1 binding to multiple comb suppressor complex 2 (Suz12-PRC2) induces oligodendrocyte formation and promotes myelin formation [59].

### Regulation of oxidative stress

Oxidative stress injury has an essential effect on SCI, which generates a large amount of oxygen free radicals, resulting in impaired cell metabolism, cell edema, and eventually apoptosis. It has been demonstrated that lncRNA CASC9, lncRNA SOX2OT, lncRNA TCTN2, and lncRNA HOTAIR are involved in oxidative stress response after SCI. LncRNA CASC9 acts as a ceRNA of miR-383-5p to regulate the LDHA-mediated Nrf2/HO-1 signaling pathway, and CASC9 overexpression can reduce SCI oxidative stress and inflammatory response [60]. It has been shown that the lncRNA SOX2OT sponge miR-331-3p in PC12 cells positively regulates Neurod1 expression and exacerbates oxidative stress, inflammatory response, and apoptosis after SCI by activating the JAK-STAT pathway [61]. In addition, miR-329-3p regulates the increase in SOD level and decrease in MDA level in LPS-stimulated PC12 cells. lncRNA TCTN2 acts as a sponge for miR-329-3p to upregulate IGF1R expression, alleviating oxidative stress, inflammatory response, and apoptosis in neurons through the miR-329-3p/IGF1R pathway [62]. High mobility group protein B1 (HMGB1) is involved in oxidative stress, inflammatory response, and apoptosis through multiple signaling pathways. lncRNA HOTAIR positively regulates HMGB1 and exacerbates oxidative stress, inflammatory damage, and neuronal apoptosis in SCI through the ROS/NF-κB signaling pathway [63].

## Limitations

This time, although there are more comprehensive statistics on the research progress about SCI in the past 5 years, there are still some limitations. First, the literature in this article is all in English, and the information of other relevant databases is not counted, and some important literature may be missed. Second, this paper mainly analyzes the research progress in the past 5 years and needs to include the relevant progress of earlier and current research. Finally, this paper only discusses SCI-related lncRNAs and does not analyze the impact of lncRNAs on SCI from other systemic diseases as a whole.

## Summary and outlook

Differential expression of lncRNA genes was found in the rat spinal cord at different times of SCI injury. LncRNA can regulate the biological process of SCI by inhibiting or promoting its expression through molecular interaction with miRNA/mRNA. However, the study of lncRNA regulation of SCI still needs to be more profound, and it is difficult to control the regulatory mechanism. lncRNA not only has a particular regulatory role in SCI but also has a similar role in other diseases. For example, one study found that lncRNA NKILA significantly alleviated the extent of cerebral infarction, brain edema, and neurological damage. It also inhibits the NF-κB pathway in cerebral infarction by inhibiting astrocytes’ inflammatory response and oxidative stress, effectively alleviating brain infarction injury [64]. In hepatocellular carcinoma, lncRNA DILC has a role in regulating inflammation. lncRNA DILC can inhibit multiple signaling pathways such as NF-κB and STAT3 to affect the proliferation of hepatocellular carcinoma stem cells by suppressing the transcription of IL-6 [65]. The lncRNA DILC induces the expression of SOCS3, which alleviates neuropathic pain by inhibiting the JAK/STAT3 pathway, and suppressor of cytokine signaling inhibitor 3(SOCS3), a member of the SOCS family, inhibits STAT3 phosphorylation, reduces the production of IL-6 and IL-1β, and is an inhibitor of the JAK/STAT3 pathway, an inflammatory signaling pathway [66]. In epileptic disorders, downregulation of lncRNA 17A promotes brain-derived neurotrophic factor (BDNF) expression, downregulating the glutamate/GABA ratio, promoting neuronal growth, and reducing neuronal excitotoxicity [67]. However, whether these lncRNAs have the same role in SCI is unknown, and their mechanism of action needs to be further investigated. LncRNAs still have great potential to be studied in the treatment of SCI. However, the research on lncRNAs is still at the stage of animal experiments, and a large number of experiments are still needed to verify the validity of lncRNAs based on the study of gene expression differences in SCI rats to predict the relevant lncRNAs.

In summary, SCI often involves multiple complex pathological processes that require significant time and effort for research. Many studies have investigated the pathogenesis of SCI and the regulatory role of related lncRNAs in SCI. However, these studies still need to form a complete regulatory network system. Further studies on the functional roles of lncRNAs and their corresponding target genes are needed to understand their complex mechanisms of action and expand the targeted therapies for SCI.

## Data Availability

Not applicable.

## References

[1] Hou Y, Liu X, Guo Y (2022). Strategies for effective neural circuit reconstruction after spinal cord injury: use of stem cells and biomaterials. World Neurosurg.

[2] Quadri SA, Farooqui M, Ikram A (2020). Recent update on basic mechanisms of spinal cord injury. Neurosurg Rev.

[3] Ding W, Hu S, Wang P (2022). Spinal cord injury: the global incidence, prevalence, and disability from the global burden of disease study 2019. Spine.

[4] Alizadeh A, Dyck SM, Karimi-Abdolrezaee S (2019). Traumatic spinal cord injury: an overview of pathophysiology, models and acute injury mechanisms. Front Neurol.

[5] Chandran R, Mehta SL, Vemuganti R (2017). Non-coding RNAs and neuroprotection after acute CNS injuries. Neurochem Int.

[6] Giordano L, Porta GD, Peretti GM (2020). Therapeutic potential of microRNA in tendon injuries. Br Med Bull.

[7] Oliviero A, Della Porta G, Peretti GM (2019). MicroRNA in osteoarthritis: physiopathology, diagnosis and therapeutic challenge. Br Med Bull.

[8] Chen FS, Tong XY, Fang B (2022). The roles of microRNAs in spinal cord ischemia–reperfusion injury. Neural Regen Res.

[9] Bossi L, Figueroa-Bossi N (2016). Competing endogenous RNAs: a target-centric view of small RNA regulation in bacteria. Nat Rev Microbiol.

[10] Wang Q, Han CL, Wang KL (2020). Integrated analysis of exosomal lncRNA and mRNA expression profiles reveals the involvement of lnc-MKRN2-42:1 in the pathogenesis of Parkinson's disease. CNS Neurosci Ther.

[11] Wang H, Meng Q, Qian J (2022). Review: RNA-based diagnostic markers discovery and therapeutic targets development in cancer. Pharmacol Ther.

[12] Xu S, Li Y, Chen JP (2020). Oxygen glucose deprivation/re-oxygenation-induced neuronal cell death is associated with Lnc-D63785 m6A methylation and miR-422a accumulation. Cell Death Dis.

[13] Derrien T, Johnson R, Bussotti G (2012). The GENCODE v7 catalog of human long noncoding RNAs: analysis of their gene structure, evolution, and expression. Genome Res.

[14] Bridges MC, Daulagala AC, Kourtidis A (2021). LNCcation: lncRNA localization and function. J Cell Biol.

[15] Wang KC, Chang HY (2011). Molecular mechanisms of long noncoding RNAs. Mol Cell.

[16] Zhou Z, Han B, Jin H (2020). Changes in long non-coding RNA transcriptomic profiles after ischemia-reperfusion injury in rat spinal cord. PeerJ.

[17] Zhou H, Shi Z, Kang Y (2018). Investigation of candidate long noncoding RNAs and messenger RNAs in the immediate phase of spinal cord injury based on gene expression profiles. Gene.

[18] Shi Z, Yuan S, Shi L (2021). Programmed cell death in spinal cord injury pathogenesis and therapy. Cell Prolif.

[19] Khan A, Shal B, Ullah Khan A (2023). Neuroprotective mechanism of Ajugarin-I against Vincristine-Induced neuropathic pain via regulation of Nrf2/NF-kappaB and Bcl2 signalling. Int Immunopharmacol.

[20] Wei S, Leng B, Yan G (2023). Targeting autophagy process in center nervous trauma. Front Neurosci.

[21] Liao HY, Wang ZQ, Ran R (2021). Biological functions and therapeutic potential of autophagy in spinal cord injury. Front Cell Dev Biol.

[22] Nguyen HX, O’Barr TJ, Anderson AJ (2007). Polymorphonuclear leukocytes promote neurotoxicity through release of matrix metalloproteinases, reactive oxygen species, and TNF-alpha. J Neurochem.

[23] Becher B, Spath S, Goverman J (2017). Cytokine networks in neuroinflammation. Nat Rev Immunol.

[24] Wang X, Fu Y, Botchway BOA (2022). Quercetin can improve spinal cord injury by regulating the mTOR signaling pathway. Front Neurol.

[25] Detloff MR, Fisher LC, McGaughy V, McGaughy V, Longbrake EE (2008). Remote activation of microglia and pro-inflammatory cytokines predict the onset and severity of below-level neuropathic pain after spinal cord injury in rats. Exp Neurol.

[26] Gaudet AD, Fonken LK (2018). Glial cells shape pathology and repair after spinal cord injury. Neurotherapeutics.

[27] Han T, Song P, Wu Z (2023). Inflammatory stimulation of astrocytes affects the expression of miRNA-22-3p within NSCs-EVs regulating remyelination by targeting KDM3A. Stem Cell Res Ther.

[28] Liu R, Li Y, Wang Z, et al. Regulatory T cells promote functional recovery after spinal cord injury by alleviating microglia inflammation via STAT3 inhibition. CNS Neurosci Ther. 2023. 10.1111/cns.14161 PMC10352886 36914969

[29] Fan B, Wei Z, Yao X (2018). Microenvironment imbalance of spinal cord injury. Cell Transplant.

[30] Singh A, Kukreti R, Saso L (2019). Oxidative stress: a key modulator in neurodegenerative diseases. Molecules.

[31] Lin J, Xiong Z, Gu J (2021). Sirtuins: potential therapeutic targets for defense against oxidative stress in spinal cord injury. Oxid Med Cell Longev.

[32] Jia Z, Zhu H, Li J (2012). Oxidative stress in spinal cord injury and antioxidant-based intervention. Spinal Cord.

[33] Albrecht J, Sidoryk-Wegrzynowicz M, Zielinska M (2010). Roles of glutamine in neurotransmission. Neuron Glia Biol.

[34] Lai TW, Zhang S, Wang YT (2014). Excitotoxicity and stroke: identifying novel targets for neuroprotection. Prog Neurobiol.

[35] Liu X, Zhang Q, Wang W (2018). Analysis of long noncoding RNA and mRNA expression profiles in IL-9-activated astrocytes and EAE mice. Cell Physiol Biochem.

[36] Liu QQ, Liu H, He ZG (2017). Differential gene and lncRNA expression in the lower thoracic spinal cord following ischemia/reperfusion-induced acute kidney injury in rats. Oncotarget.

[37] Ling X, Lu J, Yang J (2021). Non-coding RNAs: emerging therapeutic targets in spinal cord ischemia-reperfusion injury. Front Neurol.

[38] Cui SY, Zhang W, Cui ZM (2021). Knockdown of long non-coding RNA LEF1-AS1 attenuates apoptosis and inflammatory injury of microglia cells following spinal cord injury. J Orthop Surg Res.

[39] Zhao Q, Lu F, Su Q (2020). Knockdown of long noncoding RNA XIST mitigates the apoptosis and inflammatory injury of microglia cells after spinal cord injury through miR-27a/Smurf1 axis. Neurosci Lett.

[40] He X, Zhang J, Guo Y (2022). LncRNA MIAT promotes spinal cord injury recovery in rats by regulating RBFOX2-mediated alternative splicing of MCL-1. Mol Neurobiol.

[41] Liu Y, Pan L, Jiang A (2018). Hydrogen sulfide upregulated lncRNA CasC7 to reduce neuronal cell apoptosis in spinal cord ischemia-reperfusion injury rat. Biomed Pharmacother.

[42] Xia X, Niu H, Ma Y (2020). LncRNA CCAT1 protects astrocytes against OGD/R-induced damage by targeting the miR-218/NFAT5-signaling axis. Cell Mol Neurobiol.

[43] Xu Y, An BY, Xi XB (2016). MicroRNA-9 controls apoptosis of neurons by targeting monocyte chemotactic protein-induced protein 1 expression in rat acute spinal cord injury model. Brain Res Bull.

[44] Wang F, Chang S, Li J (2021). Lithium alleviated spinal cord injury (SCI)-induced apoptosis and inflammation in rats via BDNF-AS/miR-9-5p axis. Cell Tissue Res.

[45] An Q, Zhou Z, Xie Y (2021). Knockdown of long non-coding RNA NEAT1 relieves the inflammatory response of spinal cord injury through targeting miR-211-5p/MAPK1 axis. Bioengineered.

[46] Ban Y, Cui C (2020). Silencing of long non-coding RNA (lncRNA) nuclear paraspeckle assembly transcript 1 (NEAT1) protects PC-12 cells from LPS-induced injury via targeting miR-29a. Med Sci Monit.

[47] Xiang W, Jiang L, Zhou Y (2021). The lncRNA Ftx/miR-382-5p/Nrg1 axis improves the inflammation response of microglia and spinal cord injury repair. Neurochem Int.

[48] Shao M, Jin M, Xu S (2020). Exosomes from long noncoding RNA-Gm37494-ADSCs repair spinal cord injury via shifting microglial M1/M2 polarization. Inflammation.

[49] Yu Y, Zhu M, Zhao Y (2018). Overexpression of TUSC7 inhibits the inflammation caused by microglia activation via regulating miR-449a/PPAR-γ. Biochem Biophys Res Commun.

[50] Zhou J, Li Z, Wu T (2020). LncGBP9/miR-34a axis drives macrophages toward a phenotype conducive for spinal cord injury repair via STAT1/STAT6 and SOCS3. J Neuroinflamm.

[51] Dong J, Xia R, Zhang Z (2021). lncRNA MEG3 aggravated neuropathic pain and astrocyte overaction through mediating miR-130a-5p/CXCL12/CXCR4 axis. Aging (Albany NY).

[52] Li H, Xu Y, Wang G (2019). Long non-coding RNA Mirt2 relieves lipopolysaccharide-induced injury in PC12 cells by suppressing miR-429. J Physiol Biochem.

[53] Li JW, Kuang Y, Chen L (2018). LncRNA ZNF667-AS1 inhibits inflammatory response and promotes recovery of spinal cord injury via suppressing JAK-STAT pathway. Eur Rev Med Pharmacol Sci.

[54] Li Z, Li A, Yan L (2020). Downregulation of long noncoding RNA DLEU1 attenuates hypersensitivity in chronic constriction injury-induced neuropathic pain in rats by targeting miR-133a-3p/SRPK1 axis. Mol Med.

[55] Pan X, Shen C, Huang Y (2020). Loss of SNHG4 attenuated spinal nerve ligation-triggered neuropathic pain through sponging miR-423-5p. Mediators Inflamm.

[56] Hu S, Zheng J, Du Z (2020). Knock down of lncRNA H19 promotes axon sprouting and functional recovery after cerebral ischemic stroke. Brain Res.

[57] Jia H, Li Z, Chang Y (2021). Downregulation of long noncoding RNA TUG1 attenuates MTDH-mediated inflammatory damage via targeting miR-29b-1-5p after spinal cord ischemia reperfusion. J Neuropathol Exp Neurol.

[58] Lv HR (2017). lncRNA-Map2k4 sequesters miR-199a to promote FGF1 expression and spinal cord neuron growth. Biochem Biophys Res Commun.

[59] He D, Wang J, Lu Y (2017). lncRNA functional networks in oligodendrocytes reveal stage-specific myelination control by an lncOL1/Suz12 complex in the CNS. Neuron.

[60] Guan C, Wang Y (2021). LncRNA CASC9 attenuates lactate dehydrogenase-mediated oxidative stress and inflammation in spinal cord injury via sponging miR-383-5p. Inflammation.

[61] Li R, Li X, Huang Y (2021). LncRNA SOX2OT knockdown alleviates lipopolysaccharide-induced damage of PC12 cells by regulating miR-331-3p/Neurod1 axis. World Neurosurg.

[62] Liu J, Lin M, Qiao F (2022). Exosomes derived from lncRNA TCTN2-modified mesenchymal stem cells improve spinal cord injury by miR-329-3p/IGF1R axis. J Mol Neurosci.

[63] Wang Z, Long R, Yang Z (2022). lncRNA HOTAIR inhibition by regulating HMGB1/ROS/NF-κB signal pathway promotes the recovery of spinal cord function. Comput Math Methods Med.

[64] Gao W, Ning Y, Peng Y (2021). LncRNA NKILA relieves astrocyte inflammation and neuronal oxidative stress after cerebral ischemia/reperfusion by inhibiting the NF-kappaB pathway. Mol Immunol.

[65] Wang X, Sun W, Shen W (2016). Long non-coding RNA DILC regulates liver cancer stem cells via IL-6/STAT3 axis. J Hepatol.

[66] Liu Y, Feng L, Ren S (2020). Inhibition of lncRNA DILC attenuates neuropathic pain via the SOCS3/JAK2/STAT3 pathway. Biosci Rep.

[67] Zayed AA, Seleem MM, Darwish HA (2023). Role of long noncoding RNAs; BDNF-AS and 17A and their relation to GABAergic dysfunction in Egyptian epileptic patients. Metab Brain Dis.

